# Analysis of wheat SAGE tags reveals evidence for widespread antisense transcription

**DOI:** 10.1186/1471-2164-9-475

**Published:** 2008-10-10

**Authors:** Rebecca L Poole, Gary LA Barker, Kay Werner, Gaia F Biggi, Jane Coghill, J George Gibbings, Simon Berry, Jim M Dunwell, Keith J Edwards

**Affiliations:** 1 School of Biological Sciences, University of Bristol, Bristol, UK; 2 School of Biological Sciences, University of Southampton, Southampton, UK; 3 Transcriptomics Facility, School of Biological Sciences, University of Bristol, Bristol, UK; 4 School of Biological Sciences, University of Reading, Reading, Berkshire, UK; 5 Nickerson-Advanta, Station Road, Docking, Norfolk, UK

## Abstract

**Background:**

Serial Analysis of Gene Expression (SAGE) is a powerful tool for genome-wide transcription studies. Unlike microarrays, it has the ability to detect novel forms of RNA such as alternatively spliced and antisense transcripts, without the need for prior knowledge of their existence. One limitation of using SAGE on an organism with a complex genome and lacking detailed sequence information, such as the hexaploid bread wheat *Triticum aestivum*, is accurate annotation of the tags generated. Without accurate annotation it is impossible to fully understand the dynamic processes involved in such complex polyploid organisms. Hence we have developed and utilised novel procedures to characterise, in detail, SAGE tags generated from the whole grain transcriptome of hexaploid wheat.

**Results:**

Examination of 71,930 Long SAGE tags generated from six libraries derived from two wheat genotypes grown under two different conditions suggested that SAGE is a reliable and reproducible technique for use in studying the hexaploid wheat transcriptome. However, our results also showed that in poorly annotated and/or poorly sequenced genomes, such as hexaploid wheat, considerably more information can be extracted from SAGE data by carrying out a systematic analysis of both perfect and "fuzzy" (partially matched) tags. This detailed analysis of the SAGE data shows first that while there is evidence of alternative polyadenylation this appears to occur exclusively within the 3' untranslated regions. Secondly, we found no strong evidence for widespread alternative splicing in the developing wheat grain transcriptome. However, analysis of our SAGE data shows that antisense transcripts are probably widespread within the transcriptome and appear to be derived from numerous locations within the genome. Examination of antisense transcripts showing sequence similarity to the *Puroindoline a *and *Puroindoline b *genes suggests that such antisense transcripts might have a role in the regulation of gene expression.

**Conclusion:**

Our results indicate that the detailed analysis of transcriptome data, such as SAGE tags, is essential to understand fully the factors that regulate gene expression and that such analysis of the wheat grain transcriptome reveals that antisense transcripts maybe widespread and hence probably play a significant role in the regulation of gene expression during grain development.

## Background

With cereals constituting more than 60% of the world's dietary intake, the bread wheat *Triticum aestivum *is one of the most important crops in world agriculture [1,2]. Despite the high yields achieved in Europe there is still a real need to generate improved cultivars, as yield and flour quality can be dramatically affected by the environment. This need has become even greater in recent years with tightening world supplies and reduced stocks, resulting in record grain prices [3]. Over the past decade, the advent of genomic technologies has played an increasingly important role in this process. The ability to perform studies on a genome-wide scale has allowed an understanding of entire biological pathways and the complex regulatory networks of the transcriptome and has generated information that has the potential to be exploited in breeding programmes.

There are currently many tools available to measure global gene expression, perhaps the most commonly used are microarrays or GeneChips [4]. However, due to the complicated nature of the bread wheat genome; consisting of three closely related genomes (A, B and D) [5] with approximately 25% of all genes represented by at least two paralogous loci [6] and with 75% of the 16.8 Gigabases consisting of repetitive sequences [7], current microarrays have their limitations. For example, previous studies using both spotted cDNA microarrays and the Affymetrix wheat GeneChip^® ^have shown that while microarray-based platforms are capable of monitoring gene expression in polyploids, due to cross-hybridisation of related transcripts, they can be misleading as to which homoeolog/paralog-specific sequences are actually being quantified [8,9].

Serial Analysis of Gene Expression (SAGE), as described by Velculescu *et al*. [10], is now established as a powerful technique for the simultaneous, quantitative analysis of large numbers of transcripts. Since 1999 there have been numerous reports of the use of SAGE in the characterisation of the transcriptome of various plant species [11-15] including a recent report on the analysis of the developing caryopsis of wheat [16]. SAGE has several advantages over microarrays; it has a greater potential to discriminate between homoeologous and paralogous transcripts, it reveals the absolute expression values of the transcriptome allowing direct comparisons between genes, it is not limited to previously identified genes and it has no theoretical transcript detection limit [17]. SAGE therefore holds the promise of being able to identify the presence and abundance of novel transcripts including alternative spliced and/or antisense transcripts, something only possible with very specifically designed microarrays [11,16,18,19].

One of the major limitations of SAGE is that without a complete genome sequence from the species under investigation, tag annotations have to be performed using the limited sequence data available. This inevitably results in ambiguous or unassigned annotations and thus without further characterisation some data will be of limited use.

In this study we have used LongSAGE [20,21] to study gene expression in allohexaploid wheat at a developmental stage, 14 days post anthesis (dpa), when the cellular endosperm is undergoing large scale carbohydrate biosynthesis. In addition to collecting data from the transcriptome of grain derived from plants grown under standard conditions, we also obtained data from the transcriptome of grain from plants grown under relatively hot and dry conditions; conditions which are known to have a significant effect on the quantity and quality of the resulting flour [22]. To analyse the resulting tags we developed a novel approach to tag annotation, which makes best use of the publicly available sequence data. Our results show that SAGE is an effective tool to examine the wheat allohexaploid transcriptome. In addition, our investigation has shown that both alternative and antisense transcripts are present in the wheat transcriptome, sometimes at surprisingly high frequencies. Using the single copy *Puroindoline a *and *b *genes (*Pina *and *Pinb*) we have characterised the extent of these alternative and antisense transcripts and based upon these results we speculate that such sequences might play a role in grain development.

## Results and discussion

### Library production and sage tag annotation

Several previous studies have examined the transcriptome of the developing cereal grain [23-26] and more recently McIntosh *et al*. [16] used LongSAGE to study grain development in allohexaploid wheat.

Grown under typical UK conditions, UK adapted wheat varieties begin the onset of large-scale carbohydrate synthesis around 14 dpa. However, numerous studies have demonstrated that grain development is heavily influenced by environmental factors such as heat and moisture [22,23,27-29]. To obtain a wide sample of the various transcripts present during this agronomically important phase of development, we generated six LongSAGE libraries from two related commercial wheat varieties grown under two environmental conditions, as described in the methods section.

Before analysis, duplicate ditags and sequences falling below the MegaBACE quality threshold were removed. In addition, all tags were trimmed so that only the first 18 of the potential 19 bases, including the anchoring enzyme (CATG) site, were included for this analysis. The last base was removed as its presence caused a disproportionate increase in the number of distinct tags, indicating that this sequence position was unreliable. In total, 71,930 tags were sequenced across all six libraries, with individual library counts ranging from 9,786 to 13,875 (Table 1, complete dataset; GEO accession GSE12832). A good correlation was observed between the replicate libraries (average Pearson product moment 0.82), highlighting the reproducibility of the data. To our knowledge, no other study has generated such a large number of tags for a single developmental stage in wheat.

**Table 1:** Summary of SAGE libraries

Library	Total tag count	Number of Unique tags (%)	Number of singletons (%)	Number of tags with a count of >3 (cumulative count)
Xi19 (normal)	13,286	9,471 (71)	8,382 (63)	313 (3167)
Xi19 (normal) tech. rep	10,978	7,890 (72)	6,999 (64)	217 (2474)
Scorpion 25 (normal)	13,875	9,853 (71)	8,713 (63)	304 (3295)
Scorpion 25 (normal) tech. rep	9,786	4,850 (50)	4,323 (44)	527 (5393)
Xi19 (hot and dry)	12,460	7,818 (63)	6,942 (56)	260 (4136)
Scorpion 25 (hot and dry)	11,545	6,289 (54)	5,508 (48)	344 (5141)
**All libraries combined**	**71,930**	**37,615 (52)**	**31,929 (44)**	**1,883 (31,478)**

Percentages displayed are of the total cumulative tag count.

The total tag count represents 37,615 (52%) unique tags, of which 31,929 (84%), representing approximately 44% of all tags sequenced, were singletons, i.e. appear only once in the entire dataset. These values are slightly higher than those observed by McIntosh *et al*. [16], who sampled wheat grains at the same developmental stage (14 dpa) and sequenced 19,299 tags of which 40% were unique and 31% singletons. Our plants were grown in generally cooler conditions than those in this previous study and this is likely to have resulted in slower grain development and the observed differences in tag frequency. In addition, as our data comprise two, albeit closely related, varieties and two environmental conditions, it is not surprising that we see proportionately more singletons and unique tags than the equivalent library described by McIntosh *et al*. [16].

A critical step in the SAGE procedure is the annotation of the sequenced tags. Due to the large number of tags generated this procedure requires automation. The first step towards the annotation of a tag requires matching it to a previously characterised sequence e.g. an Expressed Sequence Tag (EST) or genomic sequence. A typical approach is to match tags to clustered ESTs representing putative genes (UniGenes) [30], but often these clusters are imperfect, with some genes being split into multiple clusters, while other clusters represent several genes. Such an approach could result in ambiguous tag-to-gene matches. On the other hand, one tag may match several closely related ESTs, making tag assignments to a specific EST arbitrary and resulting in a loss of information.

Once a tag has been assigned to a sequence it then has to be annotated with its gene name and putative function. Although, some sequences are already fully annotated, this is often not the case and in these circumstances BLASTX [31] searches can be employed. These problems are amplified further for an organism such as *T. aestivum*, an allohexaploid species, where a complete genome sequence is not available, often the sequence data available are poorly annotated and where few proteins have been characterised.

**Figure 1:**
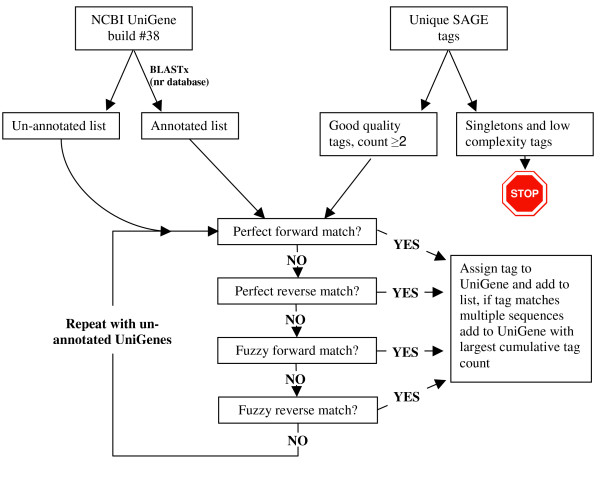
**Schematic diagram of the assignment and annotation of SAGE tags.** Each processing step was performed using a custom PERL script (Additional file 1). UniGenes are assigned annotations by BLASTX, with the UniGene sequences searched against the non-redundant (nr) protein database. Tags are preferentially assigned to UniGenes with annotations and in cases of multiple matches assigned to the UniGene with the highest cumulative frequency, to reduce redundancy within the data. Fuzzy matching tolerates up to 2 bp mismatch between the tag and the representative UniGene sequence.

**Figure 2:**
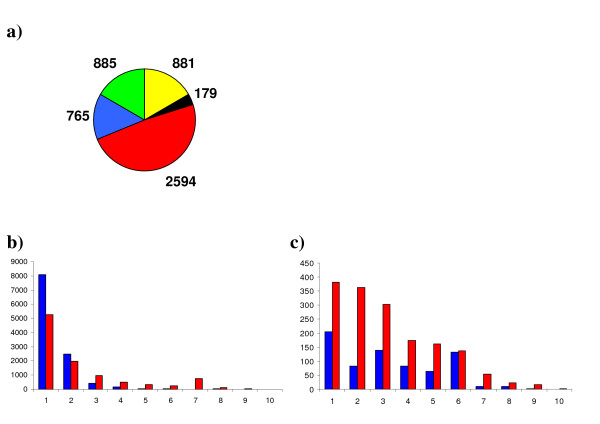
**SAGE tag classification and spatial distribution.** In total 5,304 unique tags with a count ≥2 were attempted to be assigned to a UniGene (NCBI build #38) sequence. The tags were classified into 5 categories according to the sequence alignment (a); Perfect forward matches (yellow), Perfect reverse matches (black), fuzzy forward matches (red) and fuzzy reverse matches (blue), no match to a UniGene (green). Distribution analysis of the forward (b) and reverse (c) tags across the length of the transcript was performed on total tag count data for tags with an annotation and a count ≥2 and reveals that the majority of tags are derived from the 3' most CATG site (position 1) of the respective transcripts. The perfect matched tags (blue) follow the same pattern as the fuzzy matches (red).

To overcome these challenges, we devised a novel approach to generate tag-to-gene matches, executed using custom PERL [32] scripts (Additional file 1) and described in Figure 1. The first step in our annotation process was, where possible, to assign annotation to the NCBI UniGene set build #38 (downloaded as the longest best quality EST from each of the ~38 K UniGenes). To do this, UniGene sequences were used to search the non-redundant (nr) protein database using BLASTX. As not all UniGene sequences are of the sense strand, this has the added advantage of predicting the sequence orientation. In an attempt to exclude potentially spurious tags, generated as a result of sequencing errors, only tags that were observed more than once were included. Additionally, low complexity tags (i.e. those containing microsatellites or more than 5 consecutive identical bases) were removed and this resulted in a total of 5,304 unique tags being processed. Tags were subsequently assigned to a particular UniGene using the following hierarchy; 1: Perfect tag-to-sequence match in the forward orientation. 2: Perfect tag-to-sequence match to the reverse orientation. 3: 'Fuzzy' tag-to-sequence match (a match that tolerates up to a 2 base pairs [bp] mismatch between the tag and UniGene sequence) in the forward orientation. 4: Fuzzy tag-to-sequence match in the reverse orientation. 5: No match to an EST. Initially, matches were performed against UniGenes with BLASTX annotations, as having a gene annotation adds more value to the data. In total 3,511 tags (66.2% of those processed) were assigned to an annotated UniGene. If no matches were identified for a particular tag the whole procedure was repeated for UniGenes without annotations. A further 908 tags were assigned to a UniGene in this way, resulting in a total of 4,419 unique tags (83% of those processed) assigned to a UniGene in one of the four categories; forward perfect match, forward fuzzy match, reverse perfect match, reverse fuzzy match (Figure 2a). The fully annotated dataset is available as Additional file 2.

The fuzzy matching procedure was included in our approach as it was predicted that many tags would otherwise remain un-annotated due to the incomplete nature of the sequence data available. This prediction was proved correct with only 20% of tags assigned to a UniGene by a perfect match. Fuzzy matching allows annotation assignment where sequence differences exist as a result of previously uncharacterised homoeologs, paralogs or sequencing errors within the UniGene dataset. As sequencing errors are predicted to occur once in every 100 bases, this has the potential to affect a large proportion of tag-to-UniGene assignments [33]. This effect will increase in frequency with increasing tag length (from 10% for a 10 bp tag to 20% for a 20 bp tag) and so could affect approximately one fifth of the tags within our dataset. Fuzzy matching also enables tags with no perfect match to be assigned to a closely related transcript, likely to have a similar function. This approach is of value where not all members of a multi-gene family have been sequenced, or where family members have been clustered and the gene sampled is not the same haplotype as the representative sequence. Fuzzy matching is also of use when polymorphisms exist between the wheat variety being studied and the variety from which the representative UniGene sequence was obtained. Fuzzy matching is, of course, not without its problems with the possibility of tags being assigned to the wrong gene. For example homoeologs and/or paralogs could be all assigned to the same UniGene making it impossible to investigate homoeolog/paralog-specific gene expression. This could lead to loss of information within the dataset, especially in cases where expression changes in closely related genes could cancel each other out when combined. In some cases tags may be assigned to a gene with a completely different function, but we expect these cases to be in the minority. Despite all of this a less than perfect tag-to-UniGene match is more desirable than a tag with no putative function if biological inferences are to be made from the data. Coemans *et al*. [34] also used a fuzzy matching procedure to annotate 19% of the SAGE tags generated in *Musa acuminata*, whereas 63.3% of our processed tags were annotated by fuzzy matching. This high proportion is to be expected given the lack of available sequence data and the highly complex nature of the hexaploid wheat genome.

SAGE tags should be derived from the 3' most CATG within a transcript, hence if the use of fuzzy matching is not a valid approach, then it would be expected that tags assigned in this way would be randomly distributed along the transcript. To test this, once tags were assigned to a particular UniGene their position within the sequence with respect to the 3' most CATG was determined (Figure 2b and Additional file 2 for full data set). As expected the majority of the forward perfect tags were canonical, i.e. positioned next to the most 3' CATG within the available sequence. These data also revealed that the forward perfect and forward fuzzy tags have very similar distributions along the transcript length, with the vast majority of tags being derived from the canonical position. Although this is discussed later, it is interesting to note here that there is a general trend for the reverse perfect and reverse fuzzy tags, to also be derived in the highest numbers at the 3' end of the sense transcript (Figure 2c). These observations strongly indicate that the use of fuzzy matching for annotation is a valid approach.

Interestingly, when the annotation procedure was applied to the singleton tags a similar distribution between the categories was revealed (data not shown; for the full dataset see Additional file 3).

In cases where tags matched multiple UniGenes, the tag was assigned to the UniGene with the largest cumulative tag count, to reduce redundancy within the data. Thus our 4,419 processed tags are represented by 3,268 UniGenes.

Once tags were assigned to specific UniGenes it was then possible to combine all tag counts assigned to them and after normalization to a total library tag count of 13,875 (number of tags in Scorpion25 Normal library) to investigate further the transcriptome of the wheat grain at this agronomically important phase of development.

### Gene expression at 14 dpa (tag abundance)

The grains used for this experiment were harvested at 14 dpa, a point in time which falls within the early (11–16 dpa) or 'medium milk' phase of grain filling [35]. Grain development is extremely dynamic during this period, with the initiation of storage protein accumulation, the appearance of type 'A' starch granules, division of meristematic endosperm cells, wall thickening of the cells that will form the aleurone and growth of the embryo [35]. It might therefore be expected that this wide array of developmental processes will be reflected in the diversity of SAGE tags obtained and to a large extent this expectation was met.

Forward (perfect and fuzzy) tag counts for each UniGene were combined across all six libraries and functional annotations assigned, according to the categories described by McIntosh *et al*. [16], to the most abundant. The distribution of our forward tags across the functional groups was similar to the results obtained by McIntosh *et al*. [16] (Additional file 4). Therefore this aspect of our study will not be discussed any further here (Additional file 4 contains a full description of this data), instead we have focused the rest of this analysis on the tags that often receive little attention in plant-based SAGE studies; namely alternatively spliced/polyadenylated and antisense transcripts.

### Alternative splicing/polyadenylation

Within the 2505 unique tags assessed for their position, 1332 were non-canonical. (Figure 2b). Such tags can arise by incomplete digestion with the anchoring enzyme, priming from an internal poly(A) tract or by incorrect annotation. However, several SAGE studies have reported the presence of many non-artefactual, non-canonical tags and have postulated that these represent transcripts that have been alternatively spliced or alternatively polyadenylated [13,14,19,36-39].

To investigate the presence of alternative transcripts within our forward orientation tags, we focused on the 50 most abundant UniGenes (according to forward tag count only) and removed those with internally repetitive sequences or that form part of known large multi-gene families (storage proteins and alpha-amylase inhibitors), as we could not state with confidence that a tag assigned to a non-canonical position within one UniGene was not actually a canonical tag from another family member. Within the remaining subset of data (27 UniGenes) we could find no convincing evidence for the presence of alternatively spliced transcripts despite the presence of non-canonical tags (Additional file 5). We did, however, see evidence of alternative polyadenylation within the 3' UnTranslated Regions (UTRs). This is best illustrated with the *Pina *and *Pinb *genes, selected as they are well characterised, single copy genes found only on the D genome [Genbank Accession: CR626934.1] [40-42].

**Figure 3:**
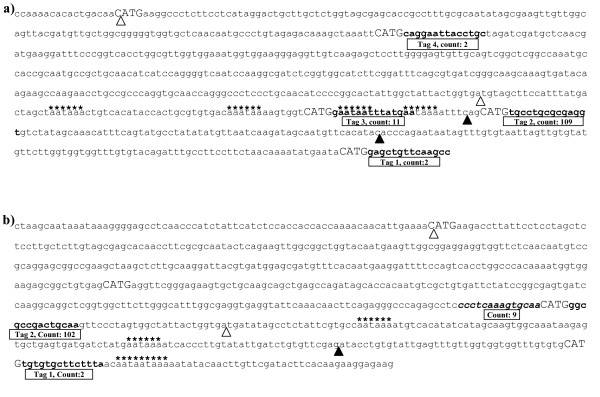
**Alignment of SAGE tags to the *Pin *genes.***Pina *(a) and *Pinb *(b) mRNA complete sequence from the *Ha *(*hardness*) locus [GenBank accession: CR626934] Chantret *et al*. [41]. All anchoring enzyme sites are denoted by upper case letters and SAGE tags in bold (reverse tags are in addition italicised), the coding sequence is delimited by open arrow heads. Putative polyadenylation signals are indicated by asterisks and the termination sites of the truncated transcripts highlighted by block arrow heads (Gautier *et al*. [43]). Cumulative tag counts across all six libraries are indicated in boxes beneath each tag. In both cases the penultimate (and non-canonical) tag has the highest frequency.

Within *Pina*, tags aligned to four of the five possible CATG sites, with only the 5' most CATG lacking a tag (Figure 3a). All four tags appear to represent alternatively polyadenylated transcripts that would not result in a truncated protein as their predicted polyadenylation signals all occur in the 3' UTR. This is consistent with Gautier *et al*. [43], who also observed *Pina *transcripts with truncated 3' UTRs.

The *Pinb *tags also revealed evidence of alternative polyadenylation (Figure 3b). Comparison with the full length *Pinb *gene [41] allowed an additional tag (tag 1) to be identified within our SAGE libraries that represented the canonical position of the full length transcript. For both *Pina *and *Pinb*, the canonical tag was not the most abundant, an observation used by others as evidence of non-canonical tag validity [18].

Ojopi *et al*., [39] also found evidence for 3' UTR alternative polyadenylation events within their *Schistosoma mansoni *SAGE libraries. They observed that truncations in the 3' UTRs often resulted in the deletion of a significant portion of the adenosine and uridine-rich elements, which target mRNAs for rapid degradation, suggesting that alternative polyadenylation plays a role in transcript stability. In addition, it has been shown that in plants, mRNAs with long 3' UTRs are more likely to be targeted for degradation by the nonsense-mediated decay pathway [44].

Both *Pina *and *Pinb *are among the most abundant sense transcripts within this data set. Such high abundance can result from either high transcription rates, low transcript decay rates or a combination of both. Thus it is plausible that the relatively low abundance of the full length mRNAs, represented by the 3' most tags, for both *Pina *and *Pinb *results in increased transcript stability.

These observations of alternative polyadenylation raise the question of how the transcription mechanism chooses between the alternative sites. The *AtFCA *gene, for example, requires a 3' end-processing protein called FY [45]. It has also been observed that antisense transcripts can drive alternative splicing and may even regulate alternative polyadenylation [46-49].

### Antisense transcripts

Consistent with other SAGE experiments, we found tags (reverse perfect and reverse fuzzy) that align to the bottom (antisense) DNA strand and thus represent putative antisense transcripts [11,12,14,16,50]. Antisense transcripts are known to occur from approximately 25–30% of all plant genes [51,52] and our data is consistent with this; of the 3,286 UniGenes assigned at least one SAGE tag, 845 (25.7%) were represented by reverse tags. Antisense transcription is typically associated with RNA interference (RNAi) mediated gene silencing, but antisense transcripts have been implicated in many other processes including occlusion of transcription and direction of DNA methylation [[53-55], reviewed in [56]]. All of these could result in a reduction of abundance of the corresponding sense transcript. However, antisense transcription has also been implicated in processes that may have little effect on sense transcript abundance such as directing alternative splicing and polyadenylation [46,48,49,55-57].

Tag counts for reverse tags (subsequently referred to as antisense) assigned to the same UniGene were combined across all six libraries and a list of the 50 most abundant antisense UniGenes compiled. Within this list we were confident that 40 UniGenes (Table 2), representing 76 antisense tags, were correctly assigned to the 'antisense' category (those lacking in annotation and an obvious polyA tail were removed) (for complete dataset see additional file 6). Each one of these 40 Unigenes was subsequently assigned to one of the nine functional groups as described by McIntosh *et al*. [16] and compared to the Unigene-based distribution of the forward (now referred to as sense) tags.

**Table 2:** Summary of 40 most abundant antisense UniGenes

**UniGene**	**Annotation**	**Functional category**	**total tag count**	**PM**	**FM**	**Sense tag(s) Present?**
gnl|UG|Ta#S17980503	no hit	Unknown	374	0	2	No
gnl|UG|Ta#S12872250	no hit	Unknown	338	0	4	No
gnl|UG|Ta#S12922882	Alpha/beta-gliadin A-II precursor (Prolamin)	Storage	238	5	5	Yes
gnl|UG|Ta#S32420068	PREDICTED: similar to rRNA intron-encoded homing endonuclease	Reproduction	190	1	3	Yes
gnl|UG|Ta#S32610130	no hit	Unknown	166	0	1	No
gnl|UG|Ta#S18010719	putative inositol-(1,4,5) trisphosphate 3-kinase [Oryza sativa]	Signalling	97	0	1	No
gnl|UG|Ta#S16057965	putative argonaute protein [Oryza sativa]	Reproduction	85	0	2	No
gnl|UG|Ta#S17985265	putative AT-hook DNA-binding protein [Oryza sativa]	Reproduction	84	0	1	No
gnl|UG|Ta#S15823985	no hit	Unknown	83	0	1	No
gnl|UG|Ta#S12923304	gamma-gliadin [Triticum aestivum]	Storage	64	5	0	Yes
gnl|UG|Ta#S26027296	UBX domain, putative [Oryza sativa (japonica cultivar-group)]	Unknown	60	0	1	No
gnl|UG|Ta#S12923123	gliadin gamma	Storage	56	3	0	Yes
gnl|UG|Ta#S12923126	low molecular weight glutenin subunit LMW-Di31 [Triticum turgidum]	Storage	48	1	1	Yes
gnl|UG|Ta#S17988646	putative glucose-6-phosphate dehydrogenase [Oryza sativa]	Metabolism	47	0	1	No
gnl|UG|Ta#S19133035	low-molecular-weight glutenin subunit group 3 type II	Storage	46	5	0	Yes
gnl|UG|Ta#S16466298	no hit	Unknown	44	0	2	No
gnl|UG|Ta#S22389847	no hit	Unknown	39	0	2	Yes
gnl|UG|Ta#S12922884	alpha-gliadin [Triticum aestivum]	Storage	35	1	0	Yes
gnl|UG|Ta#S32643313	OSJNBa0070C17.22 (CpG binding domain*)	Reproduction	34	0	2	No
gnl|UG|Ta#S13111511	wound-inducible basic protein – kidney bean	Defense	30	0	1	No
gnl|UG|Ta#S18010204	choline kinase [Oryza sativa]	Membrane	29	0	1	No
gnl|UG|Ta#S13179349	no hit	Unknown	27	0	3	No
gnl|UG|Ta#S13005586	gamma-gliadin [Triticum aestivum]	Storage	25	1	0	Yes
gnl|UG|Ta#S15880157	no hit	Unknown	24	1	0	Yes
gnl|UG|Ta#S17883810	putative serine/threonine protein phosphatase PP1 [Oryza sativa]	Signalling	23	0	1	No
gnl|UG|Ta#S12966614	putative receptor protein kinase PERK1 [Oryza sativa]	Signalling	23	0	1	No
gnl|UG|Ta#S32583944	unknown protein [Oryza sativa]	Unknown	21	0	1	No
gnl|UG|Ta#S16191894	putative wall-associated protein kinase [Oryza sativa]	Signalling	21	0	2	No
gnl|UG|Ta#S12923306	gamma-gliadin [Triticum aestivum]	Storage	21	3	1	Yes
gnl|UG|Ta#S17975314	no hit	Unknown	20	0	1	No
gnl|UG|Ta#S32572951	Nucleolar GTP-binding protein 1-like [Oryza sativa]	Signalling	20	0	1	No
gnl|UG|Ta#S22379110	putative branched-chain alpha-keto acid decarboxylase E1 beta	Cell Wall	20	0	1	No
gnl|UG|Ta#S12917789	no hit	Unknown	20	0	1	No
gnl|UG|Ta#S16228057	no hit	Unknown	20	0	1	Yes
gnl|UG|Ta#S22368491	protein phosphatase 2C, putative, expressed [Oryza sativa]	Signalling	19	0	1	No
gnl|UG|Ta#S16058509	high-molecular-weight glutenin subunit Bx17 [Triticum aestivum]	Storage	19	1	1	Yes
gnl|UG|Ta#S12932494	unknown protein; 58745–68005 [Arabidopsis thaliana]	Unknown	19	0	1	Yes
gnl|UG|Ta#S32736316	no hit	Unknown	18	1	0	No
gnl|UG|Ta#S17886389	LacZ-alpha [Shuttle vector pLPV111]	Unknown	18	1	1	No
gnl|UG|Ta#S32503514	DNA polymerase delta small subunit, putative, expressed	Reproduction	18	0	1	No

PM – Unique perfect match tags.

FM – Unique fuzzy matched tags

* – Annotation obtained by manual search

The distribution of the sense and antisense UniGenes across these nine functional groups was quite different.

Perocchi *et al*. [58] demonstrated that in microarray experiments, and indeed any transcriptome based study that includes a reverse transcription step (such as SAGE), antisense artefacts are common place. They demonstrated that approximately half of all antisense transcripts arise as a result of spurious second strand cDNA synthesis. The main cause of such spurious transcription is a hairpin loop at the 3' of the first-strand cDNA, in which case it might be expected that the tag counts for the antisense transcripts would follow that of their sense counterparts. The differences in the functional distribution of the sense and antisense tags and complete lack of tag count correlation (R^2 ^= 0.018) between the sense and antisense tags suggest that in this experiment in the most part our antisense tags have not arisen as a result of spurious antisense transcription during cDNA synthesis. Another possible cause of spurious antisense transcription is re-priming from degraded RNA fragments, this however would still be expected to result in a correlation between sense and antisense transcript abundances. A final possibility is that re-priming of the first-strand cDNA can occur from the primers used for the first- strand synthesis. As an oligo dT was used for the priming of cDNA synthesis in this experiment, it would be expected that UniGenes with antisense tags assigned to them to have polyT tracts within their gene sequence, we saw no evidence for this.

The largest functional group within the antisense UniGenes comprised 48.1% of the total tag abundance and represented those with an unknown function. This was in stark contrast with the sense tag UniGenes, where only 6.4% had no assigned function. This is perhaps not surprising as the antisense tags generally have lower abundances than the sense tags and therefore the corresponding transcripts are less likely to have been characterised.

The second most abundant group within the antisense UniGenes was the 'Storage group', which in the case of the antisense data comprised only storage proteins whereas the sense data also included the grain softness (*Gsp*) and *Pin *genes. This group represented 20.8% of cumulative antisense tag frequency, markedly different from the 65.4%, seen with the sense transcripts. Two of the nine storage proteins represented by antisense tags with cumulative abundances of 56 and 25 had low abundant sense partners with counts of 5 and 7 respectively, indicating the possibility that for these transcripts down-regulation is occurring via antisense transcription. However, six of the remaining seven transcripts within this list are also found in the 50 most abundant sense list; with the seventh appearing in the top 70. As both members of these sense and antisense transcript pairs appear to be abundant it seems unlikely that their role is sense transcript down-regulation. Therefore the antisense transcripts may serve some other purpose, such as mediating alternative polyadenylation.

The reproduction group is the third most abundant category within the antisense data. It is represented by five UniGenes and accounts for 15.5% of the total antisense transcript abundance. Each of these genes encodes a protein involved in DNA or RNA processing. The most abundant antisense transcript of this group is complementary to an rRNA homing endonuclease transcript, a protein capable of lateral transfer of introns or inteins to homologous alleles lacking the sequence [reviewed in [59]]. This group also contains an antisense transcript complementary to the *Argonaute *gene. Argonaute forms the catalytic component of the RNA-induced silencing complex (RISC), which brings about the degradation of mRNA targeted by small interfering RNAs (si-RNA) and a reduction in gene expression. Thus it appears that this mechanism of antisense gene regulation may itself be regulated by antisense transcription. A similar observation has been made for alternative splicing, where the genes involved in regulating conventional and alternative splicing are themselves heavily alternatively spliced [60].

A further antisense UniGene that may play some role in regulation of gene expression was similar to a protein with a methyl-CpG binding domain. In mammals, methyl-CpG binding proteins preferentially bind to methylated CpG dinucleotides and in doing so translate the patterns of cytosine DNA methylation into changes in transcription activity. Their role in plants is less clear-cut, as several *Arabidopsis *proteins that carry the methyl-CpG binding motif have been identified but they do not appear to bind methylated DNA [61-63]. Of the five genes in this category only one (homing endonuclease) was also represented by a forward tag, but this was only sampled five times, suggesting that these antisense transcripts may down-regulate their complementary sequences.

The signalling group makes up 7.7% of the most abundant antisense UniGenes. Within this group the most abundant antisense UniGene, putative inositol 1,4,5 trisphosphate 3-kinase (I(1,4,5)P3K), has a count more than four times higher than the next most abundant. Inositol phosphate kinases (IPKs) are reasonably well understood in animals and have been demonstrated to be important for signal transduction, for example they play a critical role in calcium homeostasis [for a review see [64]]. However, their precise roles in plants are only just coming to light. Recently, an I(1,4,5)P3K (AtIpk2β) from *Arabidopsis *was shown to promote axillary shoot branching [65]. A dual function for this protein has been demonstrated as it also has the ability to phosphorylate the carbon in the 6^th ^position, generating inositol 1,3,4,5,6 pentakisphosphate IP_5 _from I(1,3,4,5)P_4 _[65]. Xia *et al*. [66] demonstrated that AtIpk2β complements a yeast mutant lacking a transcription complex involved in arginine-metabolism-related gene expression and thus postulated that in higher plants IP3Ks may also play an important role in transcription regulation.

Numata *et al*. [67], found a subset of antisense transcripts from human, mouse, *Drosophila*, *Arabidopsis *and rice were enriched for a few ontological categories including the nucleotide binding group and suggested that "antisense-mediated regulation may occur at diverse junctions in the regulatory networks of cells". We too found nucleotide binding proteins amongst our most abundant antisense UniGenes (reproduction group), which along with those in the signalling group have the potential to affect multiple biological phenomena. In combination these two groups account for nearly a quarter (23.2%) of the most abundant antisense Unigenes and thus have potentially far reaching effects. It could be argued that when large changes are required, it would be more efficient to generate one antisense transcript that can control multiple pathways than to generate multiple individual transcripts. At the time in development investigated for this study (14 dpa) there is a transition in grain processes from cell division, expansion and differentiation towards storage protein and starch accumulation and so a more general mechanism for the down regulation of non-vital processes might be appropriate.

So far the term 'antisense transcript' has been used in its broadest sense, referring to an RNA molecule that is complementary to another mRNA. However, there are many types of antisense transcripts; they can be generated in cis- (transcription of the opposite strand within the same chromosomal region) or trans (transcribed from a different locus), they can be long or short, they can be coding or non-coding and can have numerous patterns of sequence overlap, from being completely embedded within their partner gene to having only a short overlapping region in either of the UTRs [49,55,56,67-69].

Antisense transcripts also vary in their level of sequence similarity with their target sequence, trans-encoded antisense transcripts, for example, tend to be only partially complementary in contrast to cis-encoded transcripts, which by their very nature are homologous in their overlapping range.

Although the antisense SAGE tags appear to be distributed more evenly along the length of the UniGenes than the sense (Figure 2), they are found in higher numbers at the 3' end of the sense strand (the same region from where sense tags are derived; Figure 2b and 2c). This distribution most probably reflects the diversity of the types of antisense transcripts present. For example, antisense tags that align to the 3' most CATG can arise from trans-transcription or by convergent cis-transcription of an antisense molecule with a transcription start site 3' to the end of the target gene (See Numata *et al*. [67] for sense-antisense transcript overlap classifications). To validate the observed antisense tags, we chose to perform a more detailed analysis of *Pin *gene transcription as although they did not appear in the most abundant antisense list they are single copy genes and antisense tags were detected corresponding to *Pinb *but not *Pina*.

**Figure 4:**
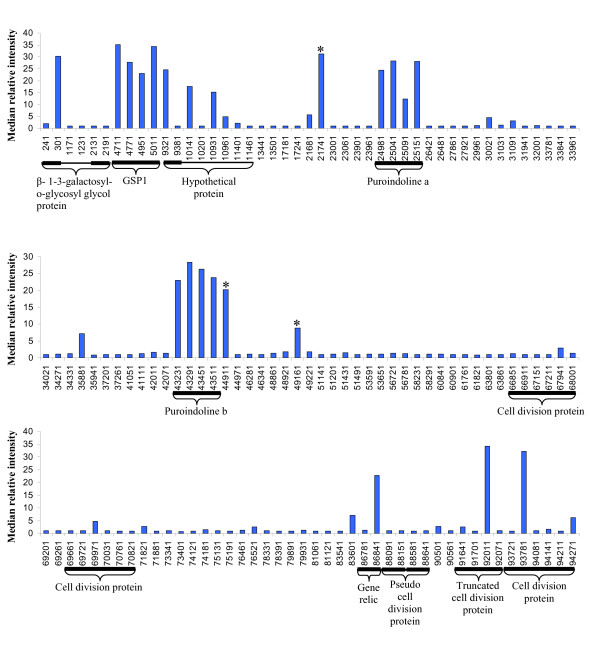
**Sense gene expression across the *Ha *Locus at 14 days post anthesis.** Each bar represents the median relative intensity of hybridisation to a 30 mer oligo. Oligo names represent the position of the first base in the oligo within the Ha locus sequence [GenBank accession CR626934]. Hybridisations were performed with cDNA from 14 dpa endosperm and revealed the ability of the microarray approach to predict the genic regions as defined in GenBank accession CR626934. Thin black lines (under the graph) indicate the gene regions with the thick black lines highlighting the coding sequences. The array also highlights areas of transcription found in the inter-genic regions (indicated by an asterisk).

Often strand specific RT-PCR is employed to assess both sense and antisense transcription. However, consistent with the findings of Haddad *et al*. [70] our extensive attempts to generate strand specific amplicons were unsuccessful (data not shown), therefore a microarray approach was employed. Initially, at least two 30-mer oligos for every predicted open reading frame (ORF) >200 bp and inter-ORF region were designed along the entire length of the *Ha *locus (Additional file 7). Hybridisation with probes derived from RNA extracted from grain at 6, 8, 10, 14, 21 and 28 dpa, revealed this to be a valid approach with the array being accurate at predicting both genic regions and novel inter-genic regions of transcription (Figure 4).

**Figure 5:**
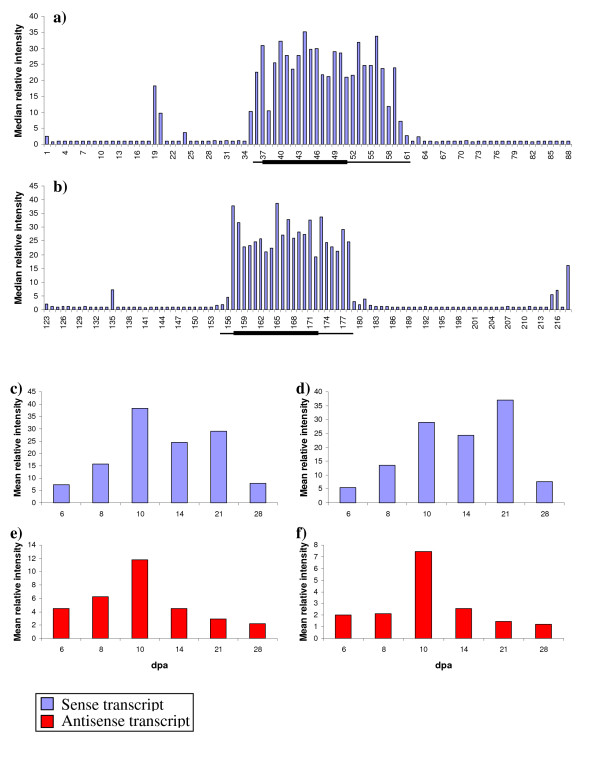
**Expression profiles of *Pina *and *b *sense (blue) and antisense (red) transcripts within the wheat endosperm. **Mean relative intensities of Pina (a) and Pinb (b) sense oligos across the tiled array, each bar represents the median relative intensity of hybridisation of cDNA from 14 dpa endosperm to a 30 mer oligo. The thin black lines under the graphs indicate the gene regions with the thick black lines representing the coding sequence. Mean relative intensities of the *Pina *sense (c), *Pinb *sense (d), *Pina *antisense (e) and *Pinb *antisense (f) transcripts were calculated over development using all anisense oligos, including both the tiled oligos and the ORF oligos. Expression of both sense and antisense transcripts peak around 10 dpa, the sense transcripts remain in abundance during the middle phase of development, whilst the antisense transcripts have declined by 14 dpa. All oligo sequences are provided in additional material 7.

To validate the presence of *Pin *antisense transcripts, tiled sense and antisense oligos were designed to cover the entire *Pina *and *b *genes and their surrounding genomic regions. Hybridisation of this array with probes derived from RNA extracted from grain at 6, 8, 10, 14, 21 and 28 dpa revealed evidence of antisense transcription for both the *Pinb *and, in contrast with the SAGE data, *Pina *transcripts. Examination of the tiled oligos confirmed that expression was largely confined to the oligos covering the transcribed regions (Figure 5a and 5b). In addition, it was apparent that while hybridisation of the sense oligos was uniform across the transcript length this was not the case with the antisense oligos (data not shown). This suggests that the antisense transcripts being measured by the arrays are transcribed in trans and thus only share interrupted regions of homology with the sense transcript. This may also explain why no *Pina *antisense SAGE tags were sampled, i.e. *Pina *antisense SAGE tags were generated but were derived from regions that do not share homology with the sense transcript and so would not have been assigned to the *Pina *UniGene using our annotation procedure. To analyse this further we combined the data generated from all oligos that covered the transcribed regions in order to compare the expression profiles of the sense and antisense tags over time (Figure 5c–f). This experiment confirmed that the sense *Pina *and *b *transcripts accumulated during early development peaking at 10 dpa, and remained at high levels during the middle phase of development (up to 21 dpa) and rapidly declined towards the end of development, a pattern similar to that observed by Gautier *et al*. [43]. The *Pina *and *b *antisense transcripts also accumulate during the early phase of development, again peaking at 10 dpa before declining in abundance. As both the sense and the antisense transcripts appear to accumulate at the same time, it seems likely that they are co-regulated. However, this pattern either means that the antisense transcripts are not down regulating the sense transcripts or that additional, as yet unknown, factors are involved in the interaction between the two. It is interesting to note that in our array experiment the antisense signal appears to decay before the sense signal, suggesting that the antisense transcript is not available to regulate the sense transcript during the later part of grain development. In this case it is difficult to interpret the role of the antisense transcript. It is plausible that the antisense *Pina *and *b *transcripts are involved in directing the observed alternative polyadenylation, which could in turn be affecting transcript stability. However, whereas we do observe different frequencies of alternative polyadenylated transcripts for both *Pina *and *b *in the different SAGE libraries the small numbers involved do not allow us to draw any statistically significant conclusions. Hence, further work is required to test this hypothesis. In addition, it must be remembered that in our array-based experiments we have used RNA derived from whole endosperm and so the possibility remains that the role of the antisense *Pina *and *b *transcripts is determined by both spatial as well as temporal regulation. Again further work using *in situ *hybridisation will be necessary to investigate this possibility.

## Conclusion

Our study has shown that detailed semi-automated analysis of SAGE-based transcriptome data can be used to extract useful information from those species for which no full genome sequence exists. Our results have also shown that in the case of species with complex polyploid genomes, such as the majority of plants, the use of fuzzy data is valid and can be used to make an important contribution to the subsequent analysis. Analysis of the dataset generated by this process has shown that for allohexaploid wheat there is no evidence for extensive alternative splicing. However, there is considerable evidence for alternative polyadenylation within the 3' UTRs. Our results also strongly suggest that the wheat transcriptome contains a large number of antisense transcripts which may have a role in gene regulation. Examination of the developmental pattern of sense and antisense transcripts showing sequence similarity to the *Pina *and *Pinb *genes suggests that the factors controlling the expression of the two may be linked. However, our results clearly show that the relationships between sense and antisense pairs can be complex and that further work is now required to examine the role that antisense transcripts play in orchestrating the transcriptome of the developing wheat grain.

## Methods

### Plant material and RNA extraction

Plants of the sibling varieties Scorpion25 and Xi19 (Nickerson-Advanta Seeds UK Ltd, Sleaford UK) were sown in five pots with 3 plants per pot and randomly placed in a glass house until just before ear emergence – split boot stage, GS45. At ear emergence plants were transferred to growth cabinets and grown under controlled conditions (Month 1: T_min_: 10°C, T_max_: 16°C, T_mean_: 14.5°C; Month 2: T_min_: 11°C, T_max_: 20°C, T_mean_: 17.6°C, with 100% field capacity irrigation) or hot and dry conditions (Month 1: T_min_: 12°C, T_max_: 21°C, T_mean_: 18.6°C; Month 2: T_min_: 13°C, T_max_: 25°C, T_mean_: 21.8°C, with 50% field capacity irrigation). Main stem ears were tagged at anthesis and whole grains (all grains from each ear) were harvested at 14 dpa. RNA was extracted from whole grains as described by Wilson *et al*. [8].

### Construction and sequencing of SAGE libraries

Libraries were constructed using 50 μg of total RNA as starting material with the I-SAGE™ Long Kit (Invitrogen, Paisley, UK) following the manufacturer's instructions except that ligations for forming concatemers and the ligation of concatemers and the vector were performed overnight. Six libraries were constructed: #1; Xi19 controlled conditions, #2; Xi19, hot and dry conditions, #3; Scorpion25 controlled conditions, #4; Scorpion25 hot and dry conditions, #5; technical replicate of library #3, #6; technical replicate of library #1.

Cloned inserts were prepared for sequencing via colony PCR: 1 μl aliquots of glycerol stock were added to 11.5 μl PCR reaction mix containing 0.05 μl M13 reverse primer (1 μg/μl; 5'-CAGGAAACAGCTATGACCATG-3') (Sigma-Aldrich, Dorset, UK), 0.05 μl M13 forward primer (1 μg/μl; 5'-CGTTGTAAAACGACGGCCAGT-3') (Sigma-Aldrich), 2 μl dNTP mix (1.25 mM), 1.25 μl 10xQiagen PCR buffer (Qiagen Ltd., Crawley, UK), 8.5 μl sdH_2_O and 0.1 μl Qiagen Hotstart Arobust *Taq *(5u/μl). The following PCR parameters were applied: 15 min @ 95°C, 35 cycles 20s @ 95°C followed by 60s @ 55°C followed by 3 min @ 72°C and finally 20 min @ 72°C.

Prior to cycle sequencing residual primers and nucleotides were removed from the PCR products by treating 3 μl of each PCR reaction with 2 μl of Exo-SAP mix (5.5 μl of exonuclease I (20u/μl), 110 μl shrimp alkaline phosphatase (1u/μl) and 115.5 μl sdH_2_O) at 37°C for 45 min. Samples were heat inactivated by incubation at 80°C for 15 min and finally cycle sequenced using the DYEnamic ET Dye Terminator Cycle Sequencing kit for MegaBACE DNA Analysis Systems (Amersham Biosciences, Buckinghamshire, UK). M13 reverse primer was used as sequencing primer.

### Tag annotation

Tags were processed and annotated using a custom PERL script (Additional file 1). Tags were matched to the non-redundant wheat UniGene set (Build #38) produced at NCBI . "Fuzzy" tag matching was performed using the PERL "aindex" function available in the String::Approx module. Significance levels for differences in SAGE counts were calculated using a further PERL script to perform randomisation tests with 100,000 permutations of the observed tag counts in the two groups being compared. Putative function was assigned to UniGenes with matching tags by performing a local BLASTX search against a copy of the non-redundant (nr) protein database available from NCBI (. An e-value cut-off of 1e^-05 ^was applied to these searches. BLAST tools were obtained from NCBI .

### Generation of the *Pina *and *b *oligo array

Two probes (30 mers), separated by 60 bp, were designed to every predicted open reading frame ≥200 bp along the *Ha *Locus (CR626934.1). In addition, tiled 30 mer probes were generated across the *Pina *and *Pinb *genes (locus coordinates: bases 23881–26520 (*Pina*) and 41041–45000 (*Pinb*)). Oligo probes (Sigma-Aldrich) were diluted in Nexterion spot solution (Schott, Jena, Germany) to a concentration of 20 ng/μl and spotted six times on Nexterion E glass slides (Schott), according to Wilson *et al*. [8].

### RNA extraction, cDNA synthesis, microarray hybridisation and data analysis

RNA was extracted from endosperm tissue at, 6, 8, 10, 14, 21 and 28 dpa. Samples were processed in duplicate (except 28 dpa and 8 dpa, where one and three replicates were processed, respectively). 20–40 μg of total RNA was treated with DNAse 1 (Promega, Southampton, UK) prior to first strand cDNA synthesis using SuperScriptII reverse transcriptase (Invitrogen, UK), in the presence of 5-(3-aminoallyl) 2'-deoxyuridine 5'-triphosphate (AA dUTP). To remove RNA, cDNAs were treated with RNAse H (Promega) and subsequently purified using a MinElute column (Qiagen Ltd.) and eluted in 10 μl water. cDNAs (targets) were labelled using either Alexafluor 555 or 647 reactive dyes (Molecular Probes Inc, Eugene, OR, USA) and were subsequently purified using Qiagen MinElute PCR purification spin columns (Qiagen).

Printed Nexterion E slides were blocked immediately prior to use, according to the manufacturer's protocol (Schott).

Labelled targets were hybridised to the arrays in hybridisation buffer (2× SSC, 0.08× SDS and 9 mM EDTA) overnight at 50°C. Following hybridisation, slides were successively washed in 2× SSC, 0.1% SDS at 50°C (2 × 5 mins), 0.2× SSC at room temperature (1 min) and 0.1× SSC at room temperature (1 min). Slides were dried in a swing-out plate rotor by centrifugation (400 *g*).

Slides were scanned and signal intensities recorded using an Axon instruments GenePix 4000B dual laser scanner and data collected using GENEPIX™ pro 4.0 software (Axon Instruments Inc., Union City, CA 94587, USA). The data were sorted by the GENEPIX™ pro 4.0 software and subsequently analysed using a series of custom PERL scripts. The expression value for each array feature was calculated as the ratio of its intensity to the median probe intensity for that array. Within-array replicate probe values were combined for the replicate arrays to produce a final set of between 6 and 18 ratios for each probe. The median of these ratios was used for subsequent analyses.

## Authors' contributions

RLP was responsible for interpretation of the data, and (along with KJE) designed the *Pin *locus array. RLP carried out the array experimentation. GLAB carried out all bioinformatic analyses. KW generated and sequenced the SAGE libraries. GB and JC helped with sequencing of the libraries. JD, SB and KJE together planned the experimental programme and contributed to data interpretation. GG helped with setting up the SAGE experimental procedure and contributed to data interpretation. RLP, GLAB and KJE wrote the manuscript.

## Supplementary Material

Additional File 1 **Zipped folder containing txt files.** PERL scripts used for SAGE data annotations and analysis Click here for file

Additional File 2 **Complete list of annotated SAGE tags (with count ≥2) and differential expression analysis.** Click here for file

Additional File 3 **Annotated list of singleton SAGE tags** Click here for file

Additional File 4 **Zip folder containing XLS and Word file.** Data and discussion about the 50 most abundant sense UniGenes. Click here for file

Additional File 5 **27 UniGenes investigated for evidence of alternative polyadenylation.** Click here for file

Additional File 6 **50 most abundant antisense UniGenes.** Click here for file

Additional File 7 **Pin array oligo sequences.** Click here for file
