# Supplementary material for: Analysis of wheat SAGE tags reveals evidence for widespread antisense transcription
Source: BMC Genomics. 2008 Oct 10;9:475. doi: 10.1186/1471-2164-9-475 (PMC2584110; doi:10.1186/1471-2164-9-475)
Supplement: Additional File 4 — Zip folder containing XLS and Word file. Data and discussion about the 50 most abundant sense UniGenes. [file 1471-2164-9-475-S4.zip › F_tag_description.doc]

Tag counts were combined across all six libraries and functional annotations assigned, according to the categories described by McIntosh *et al*. (2007), to the top 50 most abundant UniGenes. The cumulative tag count of these UniGenes is 13,307, representing almost half (43%) of all tags with a count ≥2.

Initially, 11 of the top 50 UniGenes were assigned to the ‘unknown’ group indicating they matched either an unknown protein, or had no protein match at all. However, by performing manual BLASTn searches against the NCBI non-redundant database it was possible to assign putative annotations to 5 of these two of which appear to be antisense and so were removed from this list.

Despite the lack of a full genome sequence, the inability to assign an annotation or function to 6 of the most abundant transcripts seems surprising given the rapid rise in available sequence information in wheat and further highlights the difficulties of transcriptomic research, on such an organism.

Overall the distribution of these most abundant tags across the functional groups was similar to that observed by McIntosh *et al*. at the same developmental stage (2007).

The most abundant functional group, is perhaps unsurprisingly the storage group, which in addition to the storage proteins (alpha, beta and gamma gliadins, high molecular and low molecular weight (HMW & LMW) glutenins, a globulin and an avenin-like protein) includes the transcripts encoding the grain texture proteins; grain softness protein 1 (GSP1) and puroindolines a and b (Pina and Pinb). This group also arguably represents the most important group in terms of determining grain quality and also contributes considerably to yield.

In total, just over 67.2% of the most abundant transcripts belonged to the storage group, in fact 8 out of the top 10 UniGenes fall into this category. The endosperm specific *Pina* and *Pinb* transcripts are both among the top 50, with *Pina* the more abundant of the two. This is consistent with the research of Gautier *et al*. (1994), who demonstrated that *Pina* transcript abundance is higher than *Pinb* throughout wheat grain development.

Examination of the abundance of these storage protein transcripts in both genotypes indicates that Scorpion25 has a higher level of storage protein transcripts (8 are significantly up regulated in Scorpion 25 compared to Xi19). Such a result is surprising as the determined genotype of the two lines suggested that according to 136 loci sampled via microsatellite and SNP-based genotyping the two lines are 95% similar. However, it is interesting to note that within the regions known to encode storage proteins the two lines do differ; in the region surrounding markers WMC044 on 1B, GWM190 on 5D, GWM459 on 6A and in the regions between WMC0054 and WMC0328 on 5A, and between CFA2028 and BARC108 on 7A. Hence it is logical to assume that the differences in storage protein transcript levels arise due to the different allelic content at one or more of these relatively few positions. If the differences in expression levels seen here relate to differences in protein levels and continue throughout grain development, Scorpion 25 grain might be expected to have higher levels of protein content than Xi19. This is confirmed by the UK recommended list data which suggests that while Xi19 has a protein content of 11.7% Scorpion 25 has a protein content of 11.9%. However, of interest is the fact that while Scorpion 25 is classified as a NIAB group 2 wheat (not suitable for bread-making), Xi19 is classified as a more valuable NIAB group 1 wheat. Thus, at least here, higher levels of storage protein expression are not necessarily correlated with superior milling qualities. It is possible that the difference in bread-making suitability between the two related varieties, at least in part, is due to the differences seen in the relative abundance of the *Pina* transcript. When together the wild-type alleles of *Pina* and *Pinb* (*Pina-1Da* & *Pinb-1Da*) confer a soft milling phenotype, which is less desirable for bread-making. Both Scorpion 25 and Xi19 carry Pina-1Da and the mutant Pinb-1Dc allele, which carries a Leu-60 to Pro-60 mutation and confers a hard milling phenotype (reviewed in Morris 2002). There is growing evidence that Pina plays a more important role in determining grain hardness than Pinb (Clarke and Rahman 2005, Chen *et al*., 2006, Martin et *al*. 2006). Gautier *et al.* (1994) demonstrated that Pina protein levels are mostly determined by transcription, thus the greater abundance of *Pina* transcripts in Scorpion 25 may confer a softer phenotype and result in grain of lower quality.

Comparison of the tag abundance in the libraries grown under normal conditions with those grown under hot and dry conditions suggests that abundance goes up for most of the transcripts encoding storage proteins. This is consistent with the findings of Gooding *et al*. (2003) who showed that grain grown under heat and water stress had increased protein content, despite a reduction in the length of grain fill. In order to achieve this, the rate of protein accumulation must be increased, which could be brought about by increases in transcript abundance. Such an increase in protein content would result in better quality bread-making grain. In addition to the storage protein transcripts increasing in abundance, the abundance of transcripts encoding the grain softness proteins decreases, although this is only significant for one of them. This too could result in improved grain quality.

In addition to an increase in relative abundance of the storage protein genes our data revealed a decrease in an important starch synthesis gene in the hot and dry environment relative to the control condition. These observations are consistent with previous studies and may be largely explained by asymmetric development of plants in the two environments (Tashiro and Wardlaw, 1989, Altenbach *et al.*, 2002, Altenbach *et al.*, 2003, Dupont and Altenbach, 2003).

The second most abundant group (defence group) isn’t directly involved with the physical changes that can be seen occurring during this phase of grain development, but clearly performs a vital role, making up 15.9% of the total transcript abundance. This group is dominated by alpha-amylase-inhibitors, one of which (dimeric alpha-amylase inhibitor) is one of the top 10 most abundant transcripts. Alpha amylases are often annotated as storage proteins also, but to remain consistent with McIntosh *et al*. (2007), they form part of the defence group.

Wheat alpha-amylase- and trypsin/alpha-amylase inhibitors, have been demonstrated to inhibit insect and microbial proteases and amylases, suggesting a role in pathogen defence (Gomez et al., 1989, Feng *et al*., 1996, reviewed in Franco *et al*., 2002, Islamov and Fursov, 2007). The transcript encoding the chloroform/methanol soluble (CM) protein within our list, CM17, encodes a subunit of a D genome tetrameric alpha-amylase inhibitor (Gomez et al., 1989) and thus also has a role in plant defence. Serpins too have been implicated in the plant pathogen defence response, with inhibitory activities against mammalian, plant and microbial proteinases (Østergaard *et al*., 2000). Some serpins however, may serve a dual role, also acting as a storage protein. The evidence for this comes from their glutamine-rich reactive centres which resembles similar regions in the prolamin storage proteins (Østergaard *et al*., 2000).

With such large amounts of resources being directed towards the grain, it is easy to see why so much of the transcriptome is dedicated to protecting the grain from invading pathogens, such as bacteria, fungi and insects. This gene group increases in prominence over grain development, dominating the transcription machinery by 40dpa (McIntosh *et al*. 2007). By this time the defence grouping also includes genes that respond to stress, most likely to protect the grain as it dries. Our data suggests that several of the UniGenes in this group respond to stress, with transcript abundances higher for the dimeric alpha-amylase inhibitors, CM17, serpin and type V thionins in the hot and dry environment compared to the normal environment. This is conisitent with Horváth-Szanics *et al*., who demonstrated that alpha-amylase inhibitors respond to drought, suggesting they may later play an additional role during dry-down.

The third most abundant group comprises genes involved in metabolism and starch synthesis, accounts of 7.3% of the most abundant transcripts and includes the final transcript of the top 10 list; pyruvate kinase. Pyruvate kinase (PK) is one of the regulatory proteins of the glycolytic pathway and the presence of its transcript amongst the most abundant reflects the huge energy requirements of the plant at this active stage of development. There are two PK isozymes, the plastidic (PK_p_) and cytosolic (PK_c_) pyruvate kinases. While the main purpose of glycolysis in both the cytosol and chloroplasts is to generate ATP, reductants and carbon skeletons for biosynthesis, there are subtle differences between the two. Cytosolic glycolysis for example is proposed to provide plants with the requisite metabolic options to aid development and acclimation to environmental stresses (for a review of glycolysis see Plaxton 1996). Interestingly, the transcript identified here is predicted to encode the cytosolic form, which would seem logical at this ‘dynamic’ stage in the grain’s development. Also, PK_c_ transcript abundance is higher in Scorpion 25 relative to Xi19, which may be as a result of increased energy requirements due to synthesis of greater amounts of storage proteins.

Although the storage group is likely to be the most important in determining grain quality, this group also includes genes involved in another process of great importance in determining grain quality and yield; starch synthesis (for a review of starch synthesis see James *et al*., 2003). Starch synthesis is especially important in determining grain yield, making up 65-75% of final grain weight. Of the 27 rice starch synthesis genes (or gene families) described by Zhu *et al*. (2003), we identified 16 within our libraries. Ignoring the alpha-amylase inhibitors described previously and categorized as defence related, the most abundant transcript identified encodes ADP-glucose pyrophosphorylase. ADP-glucose pyrophosphorylase catalyses the rate-limiting step of starch synthesis and thus has a regulatory role of the biosynthetic pathway. Only one other transcript (an endosperm beta-amylase), was amongst the 50 most abundant, a sucrose synthase was ranked 95 and the remainder had very low abundancies. Although seemingly counterintuitive given the rate of starch accumulation at this time in development, low expression of the majority of the starch biosynthetic genes was also observed by McIntosh *et al*. (2007), who argued this was due to the stability of the enzymes and their ability to catalyse multiple reactions. This raises and important issue that should always be kept in mind for studies of this kind: low transcript abundance does not mean low importance, or even necessarily low protein levels or activities. Some proteins may be stable with low turnover rates, or have high activities, in which case high transcription rates may not be necessary.

The abundance of only two of the transcripts in this group was affected by the hot and dry environmental regime. These encode the enzymes ADP-glucose pyrophophorylase (small subunit) and the large sub unit of ribulose-1,5-bisphosphate carboxylase/oxygenase (RuBisCO) and are both down regulated in the hot and dry environment. As these enzymes regulate the starch synthesis pathway and Calvin cycle, respectively, it is plausible that changes in their transcript abundance could have a knock-on effect, affecting the output of the both processes. A BLASTn search of the UniGene revealed the ADP-glucose pyrophosphorylase tags to be derived from the small sub unit of ADP-glucose pyrophosphorylase, which has been shown to play an important role in the allosteric regulation of the tetrameric enzyme (Cross *et al*., 2004). In concordance with our results, Hurkman *et al*., (2003) demonstrated that high temperatures during grain fill in wheat result in lower transcript abundance (that was independent of the changes in compression of development) of a number of starch biosynthetic genes, including ADP-glucose pyrophosphorylase, which was accompanied by reduced starch synthesis, grain weight and yield. Other studies have also demonstrated a link between ADP-glucose pyrophosphorylase activity and starch content or yield (potato; Stark *et al*., 1992, and rice; Smidansky *et al.*, 2003). Thus it seems likely that changes in the transcript abundance of ADP-glucose pyrophosphorylase is at least partially responsible for environmental fluctuations in wheat grain yield.

The remaining 4.0% of transcript abundance is divided between 4 groups: protein synthesis (0.4%), reproduction, including DNA binding proteins (1.8%), membrane and transport proteins (0.5%) and finally signaling (0.5%), each represented by only one or two transcripts.

**References in addition to those cited in the manuscript:**

Chen F, He ZH, Xia XC, Xia LQ, Zhang XY, Lillemo M, Morris CF: **Molecular and biochemical characterization of puroindoline a and b alleles in Chinese landraces and historical cultivars.** *Theoretical Applied Genetics* 2006, **112**:400-409.

Clarke B, Rahman S: **A microarray analysis of wheat grain hardness.** *Theoretical Applied Genetics* 2005, **107**:1463-1468.

Cross JM, Clancy M, Shaw JR, Greene TW, Schmidt RR, Okita TW, Hannah C: **Both subunits of ADP-Glucose Pyrophosphorylase are regulatory.** *Plant Physiology* 1994, **135:**137-144.

Dupont FM, Altenbach SB: **Molecular and biochemical impacts of environmental factors on wheat grain development and protein synthesis.** *Journal of Cereal Science* 2003, **38:**133–146.

Feng GH, Richardson M, Chen MS, Kramer KJ, Morgan TD, Reeck GR: **alpha-Amylase inhibitors from wheat: amino acid sequences and patterns of inhibition of insect and human alpha-amylases.** *Insect Biochemistry and Molecular Biology* 1996, **26:**419-426.

Franco OL, Ridgen DJ, Melo FR, Grossi-de-sá MF: **Plant alpha-amylase inhibitors and their interaction with insect alpha-amylases, structure, function and potential for crop protection.** *European Journal of Biochemistry* 2002, **269**:397-412.

Gomez L, Sanchez-Monge R, Garcia-Olmedo F, Salcedo G: **Wheat tetrameric inhibitors of insect alpha-amylases: Alloploid heterosis at the molecular level.** *Proceedings of the National Academy of Sciences USA* 1989, **86:**3242-3246.

Horváth-Szanics E, Szabó, Z, Janáky T, Pauk J, Hajós G: Proteomics and and emergent tool for identification of stress-induced proteins in control and genetically modified wheat lines. Chromatographia Supplement 2006, **63:**S143- S147.

Hurkman WJ, McCue KF, Altenbach SB, Korn A, Tanaka CK, Kothari KM, Johnson EL, Bechtel DB, Wilson JD, Anderson OD, DuPont FM: **Effect of temperature on expression of genes encoding enzymes for starch biosynthesis in developing wheat endosperm.** *Plant Science* 2003, **164:**873–881.

Islamov RA, Fursov OV: **Bifunctional inhibitor of alpha-amylase/trypsin from wheat grain.** *Applied Biochemistry and Microbiology* 2007, **43:**379-382.

James MG, Denyer K, Myers AM: **Starch Synthesis in the cereal endosperm.** *Current Oppinion in Biology* 2003, **6:**215-222.

Martin JM, Meyer FD, Smidansky ED, Wanjugi H, Blechl AE, Giroux MJ: **Complementation of the Pina (null) allele with the wild type *Pina* sequence restores a soft phenotype in transgenic wheat.** *Theoretical Applied Genetics* 2006, **113:**1563-1570.

Østergaard H, Rasmussen SK, Roberts TH, Hejgaard J: **Inhibitory serpins from wheat grain with reactive centres resembling glutamine-rich repeats of prolamin storage proteins.** *The Journal of Biological Chemistry* 2000, **275:**33272-33279.

Plaxton W: **The organization and regulation of plant glycolysis.** *Annual Review of Plant Physiology and Plant Molecular Biology* 1996, **47:**185-214.

Smidansky ED, Martin JM, Hannah LC, Fischer AM, Giroux MJ: **Seed yield and plant biomass increases in rice are conferred by deregulation of endosperm ADP-glucose pyrophosphorylase.** *Planta* 2003, **216**:656-664

Stark DM, Timmerman KP, Barry GF, Preiss J, Kishmore GM: **Regulation of the amount of starch in plant tissues by ADP glucose pyrophosphorylase.** *Science* 1992, **258:**287-292.

Tashiro T, Wardlaw IF: **A comparison of the effect of high temperature on grain development in wheat and rice.** *Annals of Botany* 1989, **64:**59-65

Zhu T, Budworth P, Chen WQ, Provart N, Chang HS, Guimil S, Su WP, Estes B, Zou GZ, Wang X: **Transcriptional control of nutrient partitioning during rice grain filling.** *Plant Biotechnology Journal* 2003 **1:** 59-70.
